# Adding insult to injury: Ship groundings are associated with coral disease in a pristine reef

**DOI:** 10.1371/journal.pone.0202939

**Published:** 2018-09-12

**Authors:** L. J. Raymundo, W. L. Licuanan, A. M. Kerr

**Affiliations:** 1 The Marine Laboratory, University of Guam, Mangilao, Guam, United States of America; 2 Department of Ecology and Evolutionary Biology, Cornell University, Ithaca, New York, United States of America; 3 Br. Alfred Shields FSC Ocean Research Center and Biology Department, De La Salle University, Manila, Philippines; Havforskningsinstituttet, NORWAY

## Abstract

In 2013, the remote Tubbataha Reef UNESCO World Heritage Site, in the western Philippines, experienced two ship groundings within four months: the USS *Guardian* (USSG), a US military vessel, and the *Min Ping Yu* (MPY), an illegal Chinese fishing vessel. Here, we present the results of coral disease assessments completed two years post-grounding and recovery patterns monitored annually within these grounding sites. Site assessments were undertaken in three distinct zones: ‘ground zero’, where reef was scoured to its limestone base by direct ship impact; the ‘impact border’, containing surviving upright but damaged, abraded and fragmented colonies injured during ship movement; and undamaged ‘control’ sites, remote from the ship groundings but located on the same atoll. Coral diseases were dominated by white syndromes, and prevalence was an order of magnitude higher within the impact border zones than within the other zones two years after the events. Hard coral cover has steadily increased at a mean rate of 3% per year within the scoured USSG site at a rate comparable to control sites. In contrast, recovery has been negligible within the rubble-dominated MPY site, suggesting that substrate quality strongly influenced recovery processes such as recruitment, as larvae do not survive well on unstable substrates. Long-term recovery trajectories from these two grounding events appeared strongly influenced by movement of the ship during and after each event, and site-specific wave-influenced persistence of rubble and debris. High prevalence of coral disease among damaged but surviving colonies two years post-grounding suggested long-term impacts which may be slowing recovery and creating localized pockets of higher persistent disease prevalence than that of the surrounding population.

## Introduction

**Fig 1:**
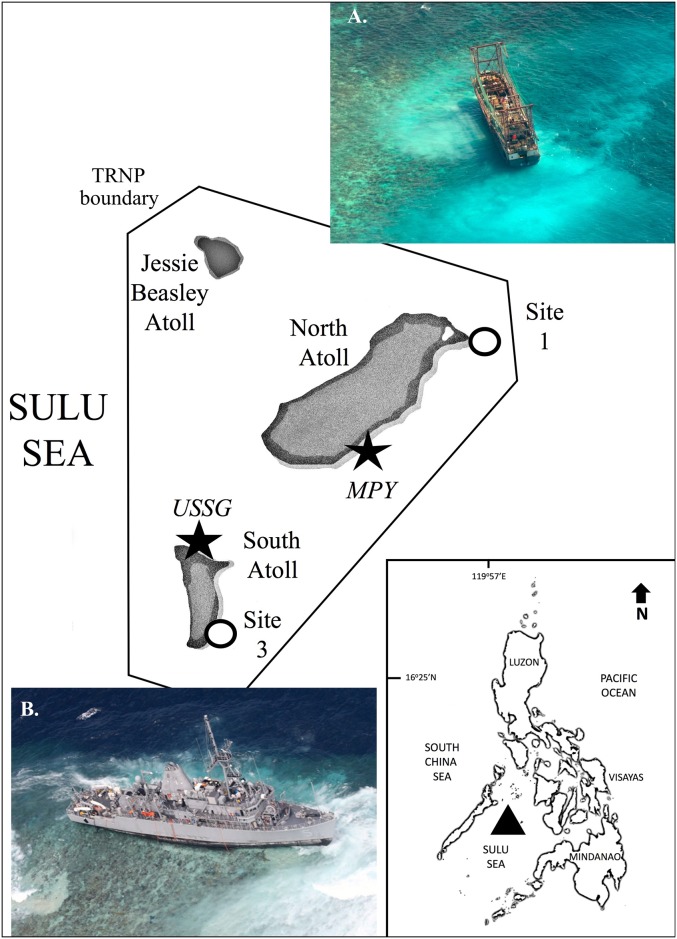
Map of Tubbataha Reefs Natural Park (TRNP), showing grounding (star) and control (open circle) sites, with inset of the Philippines, showing the position of TRNP (triangle). North Atoll: A. *Min Ping Yu*: http://gcaptain.com/chinese-fishing-vessel-runs-aground-on-tubbataha-reef-in-philippines/. Date accessed: 12.12.17; Control Site = Site 1. South Atoll: B. *USS Guardian*: http://globalbalita.com/wp-content/uploads/2013/01/USS-Guardian-grounded.3.jpg Date accessed: 12.12.17. Control Site = Site 3. All photos are public domain and are therefore not subject to copyright law.

In 2013, two ship groundings occurred within four months on the remote and largely inaccessible atolls of the Tubbataha Reefs Natural Park (TRNP), in the western Philippines. On January 17, a US Navy minesweeper, the USS *Guardian* (USSG), ran aground on the northern tip of the southern atoll, damaging 2,346 m^2^ of consolidated reef along the reef crest [1]which, prior to the grounding, supported an average of 38% live coral cover [2]. On April 8, the Chinese fishing vessel, *Min Ping Yu* (MPY), ran aground along the southeastern margin of the northern atoll, damaging an additional 3,902 m^2^ of coral garden reef community [1]. The shallow communities of both grounding sites were scoured to the limestone bedrock and large debris fields were created by ship movement. The USSG site was exposed to prevailing-wind swell and it took 72 days for the ship to be extracted. Rubble and debris were, however, quickly transported off the site by wave action. In contrast, the MPY site was relatively sheltered gentle slope, the boat was extracted in 11 days, but hit the reef three times before coming to rest (Fig 1). These conditions resulted in the persistence of rubble and debris within the grounding footprint.

Ship groundings are acute, catastrophic events for corals that create shifting debris fields which can extend the damage footprint and impede recovery. Coral recruits show low survival on unstable rubble substrates [3,4] and may also be impacted by contaminants deposited by a wreck [5]. These conditions provide opportunities for settlement of non-reef building species, resulting in localized phase shifts or blooms [6,7]. Sudden loss of topographic complexity impacts fish assemblages and may thus drive a shift in trophic structure which can take decades to return to pre-grounding levels [7]. However, to our knowledge, no studies have, as yet, investigated an association between ship grounding events and coral disease.

Our understanding of the processes that influence coral disease ecology has expanded due to the increased attention diseases have received in recent decades as drivers of reef degradation [8–10]. Correlative experimental evidence demonstrates that prevalence and/or severity of certain diseases are associated with a variety of environmental influences that include nutrient enrichment [11–14], predation [15–17], warming sea surface temperatures [18], coral bleaching [18–21], algal abrasion [22], host abundance [23], fouling by plastic debris [24], and physical injury [25–28].

Physical injury to corals can facilitate the establishment of infectious disease by damaging host tissues, thus stressing the host and creating entry wounds for opportunistic pathogenic microbes. Corals have evolved a suite of responses to this threat with immediate, short-term (minutes to days) immune strategies that include clotting, inflammation, immune-cell proliferation, and melanin production [29,30]. Wound repair involves allocation of energy to tissue regeneration and reattachment to the substrate or fusion to adjacent branches, which may take days to weeks, depending on wound size [31,32]. However, when an acute catastrophic event occurs resulting in massive, widespread injury among a population of corals, their immune capacity and the speed at which they can heal may be inadequate to prevent microbial infection. Infectious microbes may spread quickly within a population of stressed and physically damaged hosts. This could potentially trigger a disease outbreak resulting in longer-term consequences.

The TRNP is one of the most pristine and intact reefs in the Philippines, partly due to its inaccessibility from inclement seas nine months of the year. Covering a reef area of 130,028 ha, it consists of two main atolls (North and South; see Fig 1) and was declared a UNESCO World Heritage Site in 1993. The grounding incidents were the first of their kind in this atoll, resulting in catastrophic physical damage at two sites, within months of each other. This report summarizes responses of the coral community to these events within this pristine reef system. We document coral recovery processes and examine coral disease prevalence within the impact footprint and in intact communities remote from the grounding sites. We hypothesized that we would see evidence of higher disease prevalence among corals within the grounding footprints than among those within undamaged control sites, which we posit would show very low disease prevalence relative to less pristine reefs elsewhere in the Philippines [33,34].

## Methods

### Reef recovery

For all work presented in this paper that was conducted within the TRNP, permission was granted by the Tubbataha Management Office (TMO), which is the agency responsible for managing the Park. Weather permitted brief access by a biological team to the USSG grounding site four months after the incident, when two distinct zones were readily distinguishable within the grounding footprint: a ‘ground-zero’ zone where the ship hull had initially hit, characterized by reef scoured to limestone bedrock and no standing coral; and a ‘impact-border’ zone, where post-grounding ship movement and removal had occurred, characterized by shifting rubble and breakage, toppling and abrasion of standing corals. In May 2014 (17 mo post-grounding for the USSG and 15 mo post-grounding for the MPY), one 4 m x 4 m fixed monitoring plot was established in each of these two zones and a third plot in an adjacent undamaged control site 40 m from the impact-border, to monitor recovery processes. These plots have been monitored annually during the summer calm season when site access is possible.

In contrast, within the MPY site, ‘ground-zero’ and ‘impact border’ zones were less distinguishable due to the persistence of rubble, fractured colonies, and debris. Therefore, recovery monitoring plots were set up on the fragments of corals left behind by the vessel. One plot was established on the piles of small fragments and rubble (20–40 cm diameter; the ‘rubble zone’); a second plot was established on reef pavement containing large fragments of corals shattered by the rudder (~ 1 m diameter; the ‘large fragments zone’) (see S1 Fig). Lastly, a single control plot was demarcated within the undamaged community 35 m distant to the impact area. These plots were established simultaneously with those at the USSG site.

To monitor hard coral recovery, fixed plots were photographed with at least 50% overlap between adjacent images, following the approach of van Woesik et al. [35]. This resulted in a minimum of 90 images, each covering a 1 m x 1 m area, per 4 m x 4 m plot per year. For each monitoring year, a randomly chosen subset of 30 images per plot was scored using the CPCe program [36]. CPCe generated ten randomly-distributed points per image that were identified by category (live hard coral, rubble, dead coral, pavement); all live corals were identified to genus. Post hoc power analysis of per frame data per year showed that the probability of incorrectly accepting a false null hypothesis (i.e., incorrectly concluding that hard coral cover has not changed) is less than 0.2 with an alpha of 0.05 and a desired ability to detect a 6% absolute change in hard coral cover. We thus considered our n of 30 images to be adequate to detect change within and between plots and monitoring years.

### 2015 two-year post-grounding disease surveys

Coral disease surveys were conducted two years after the grounding incidents, at the request of the Tubbataha Management Office (TMO) to establish a baseline disease assessment for the Park. Undamaged control sites were selected from among regularly monitored sites on each atoll that housed previously established permanent transects of the TMO (Sites 1 and 3; Fig 1). Replicate 20 m x 1 m belt transects that followed the 4 m depth contour (Site 1: *n* = 2 transects and 1,339 colonies censused; Site 3: *n* = 3 transects and 529 colonies censused). At the grounding sites, hull scars were clearly visible two years after the events, and much of the unconsolidated debris was gone. Because each ship grounded in a different position relative to the shallow forereef and reef crest, grounding site transects could not be positioned similarly to that of the control sites. Rather, replicate 10 m x 1 m transects were positioned between 3 m and 5 m depth, within each of the ‘ground-zero’ and ‘impact-border’ zones, to allowed for specific placement of replicate transects within each damage zones. Thus, while the position of these transects was dictated by the shape of the individual grounding site footprint, the depth, reef zone (i.e., shallow forereef), and amount of reef area surveyed were comparable to those of the undamaged control sites. The number of transects was determined by the width of each zone (*USSG*: ground-zero: *n* = 9; west impact-border: *n* = 3; east impact-border: *n* = 4; *n* = 285 colonies censused; *MPY*: ground-zero: *n* = 3; lagoonal impact-border: *n* = 3; seaward near impact-border: *n* = 3; seaward far impact-border: *n* = 3; *n* = 274 colonies censused).

Along each belt, the coral community was surveyed as follows: all colonies were identified to genus and binned into pre-established size classes (SC), based on maximum colony diameter: SC1: 1–10 cm; SC2: 11–30 cm; SC3: 31–60 cm; SC4: 61–100 cm; SC5: 100–200 cm; SC6: ≥200 cm. These size classes were developed in conjunction with previous assessments and monitoring programs [13, 37–38], based on average colony sizes of common taxa. Each colony was visually inspected and assessed for the following health impacts: coral diseases (white syndromes, black band, brown band, skeletal eroding band, growth anomalies, ulcerative white spots; see S2 Fig for examples of these diseases), signs of physical injury (colony fragmentation, abrasion/ shearing, or fracture; see S3 Fig for examples of these injuries), persistent partial mortality of unknown cause. Persistent partial mortality was defined as a previous assault to a colony resulting in tissue loss with no resheeting, the cause of which was not discernible. These lesions were marked by an area of bare skeleton fouled by turf algae and other encrusting organisms, and an eroded border between the lesion and healthy tissue which, in some cases, displayed a pigmentation response of the host coral but no signs of active tissue loss or resheeting.

### Data analysis

Prevalence was calculated for each impact (disease or damage) observed using the standard formula [(no. colonies with impact *X*) / (total number of colonies)] * 100. Types of physical damage (colony toppling, fracture, abrasion/ shearing, and breakage) were pooled for analysis of prevalence between surveyed zones. Prevalence of individual diseases, damage, and partial mortality were tested separately.

G-tests were used to examine differences in white syndrome prevalence between zones and grounding sites, with a Williams’ correction for pairwise comparisons post-hoc, to determine which comparisons were significant. Bonferroni multiple comparisons corrections were calculated to determine the appropriate p-value when testing for differences between zones (USS Guardian: p < 0.05/6 = 0.0083; Min Ping Yu: p < 0.05/10 = 0.005). ANOVAs, with a Scheffe post-hoc test, were used to examine prevalence differences in partial mortality and physical damage between zones and grounding sites. Data sets that did not meet the assumptions of normality (Kolmogorov’s test) and homoscedasticity (Levene’s Test) were appropriately transformed. However, for data that were slightly non-normal but homoscedastic, ANOVA was used as this test is considered robust to even moderate deviations from normality [39]. A stepwise multiple linear regression was used to examine potential associations between disease prevalence and coral colony density, breakage, and partial mortality, after affirming that collinearity did not exist among the three independent variables. Increment of determination tests then examined which individual predictors were significantly contributing to the total r^2^ value; nonsignificant predictors were then removed from the equation. A Kruskal-Wallis test was used to examine change in percent hard coral cover between monitoring years, as data could not be normalized with transformation.

## Results

### Reef recovery

**Fig 2:**
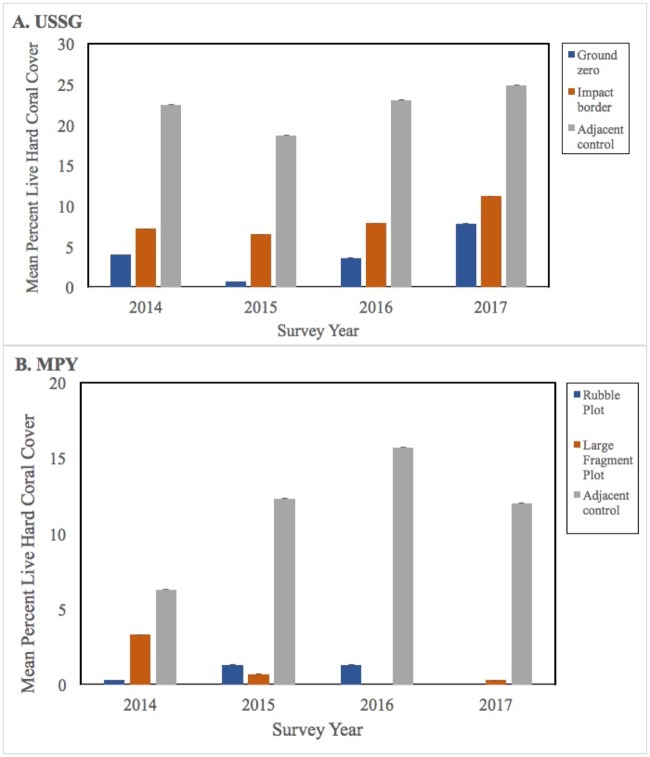
Percent live hard coral cover in monitored plots, determined annually from 2014 to 2017, based on three fixed plots within each of the two grounding sites. (A) USS *Guardian*; (B) *Min Ping Yu*. Mean +/- SEM.

The USSG hit perpendicular to the reef front, creating the ground-zero scar, then swiveled and came to rest parallel to the reef front, creating the impact border zones on either side of the initial hull scar (refer to Fig 1). One year post-grounding, the undamaged control plot showed five times more live coral cover (22.3% ± 14.9%) than the ground zero plot (4.0% ± 6.2%) and three times more coral than the impact-border plot (7.2% ± 10.5%). Coral recruits, mainly *Pocillopora*, were observed within the plots during this survey, but did not persist into subsequent monitoring periods and rapid turnover has continued to the present. Hard coral cover (HCC) steadily increased from 2014 onwards (Fig 2a), ranging from 1% to 4% per year per plot (Table 1) and grounding site plots followed similar growth trends to control plots (*H*_adj_ = 1.04; *p* = 0.406). An exception to this trend was a visible decrease in HCC in all USSG sites in 2015, though growth has been positive since that time (Table 1, Fig 2a, S1 Table). Increase in cover was largely driven by resheeting and growth of remaining corals, as recruits showed high turnover. By 2017, long-lived taxa, particularly merulinids, were growing to appreciable size.

**Table 1:** Mean (± SE) percent rate of change in hard coral cover between monitoring years in three plots within each of two grounding sites.

Plot	Rate of Change	Rate of Change	Rate of Change
	2014–15	2015–16	2016–17
*USSG* Ground Zero	-3.0 ± 0.01	3.0 ± 0.01	4.0 ± 0.02
Impact Border	-1.0 ± 0.03	1.0 ± 0.03	3.0 ± 0.03
Adjacent Control	-4.0 ± 0.05	4.0 ± 0.05	2.0 ± 0.05
*MPY* Rubble Zone	1.3 ± 0.01	0.0	-1.3 ± 0.01
Large Fragment Zone	-2.7 ± 0.02	-0.7 ± 0.0	0.3 ± 0.0
Adjacent Control	5.9 ± 0.04	3.5 ± 0.05	-3.5 ± 0.04

The MPY, having run aground in three spots within the impact footprint, created a single discernible ground-zero hull scar where it finally came to rest (Fig 1). The extensive rubble impact border zone created an impact footprint almost twice the area than that of the USSG (3,902 m^2^ vs. 2,346 m^2^), and has persisted over time. One year post-grounding, coral cover in the undamaged control plot (6.5% ± 0.1%) was twice that of the large fragment plot (3.4% ± 0.1%) and 20 times that of the fine rubble plot (0.3% ± 0.02%) (Fig 2b). The rubble and fragment plots have shown significantly less to zero increase in hard coral cover over time (*H*_adj_ = 18.83; *p* < 0.001), suggesting that persistent unconsolidated substrate is hampering recovery at this site.

### 2015 two-year post-grounding coral disease assessment

While undamaged control sites (Sites 1 and 3) displayed high colony densities and rare to no disease and physical injury, there was evidence of acute coral disease (S2 Fig), persistent partial mortality, and physical injury (S3 Fig) in both grounding sites two years after the events (Table 2, S2 Table). White syndromes (WS) were the most prevalent diseases, observed in all sites; other diseases were rare to absent. Signs of physical damage were very low to zero in control sites and highly variable in the grounding sites. Ground zero zones contained very little coral, most of which were intact post-grounding recruits. Impact borders contained numerous adult corals that showed higher disease, partial mortality, and physical injury prevalence.

**Table 2:** Summary of impacts to coral health assessed from two ship grounding sites: *Min Ping Yu* (MPY) and USS *Guardian* (USSG). Mean prevalence (± SD) of the following diseases is presented: WS = white syndromes; BBD = black band disease; BrB = brown band disease; SEB = skeletal eroding band; UWS = Ulcerative white spot; GA = growth anomalies. Damage includes breakage, toppling, abrasion. Partial Mort refers to mortality with an unknown cause and without current tissue loss. Mean ± SD reported.

Site	Mean Colony No./10m^2^	WS	BBD	BrB	SEB	UWS	GA	Total Disease	Partial Mort	Physical Damage
USSG Ground Zero	8 ± 3	2.9% ± 7.9%	0.05% ± 0.07%	0	0	0	0	2.9% ± 7.9%	0.2% ± 0.4%	7.8% ± 11.4%
USSG Impact Border	31 ± 9	13.5% ± 5.4%	0	0	0	0	0	13.5% ± 5.4%	27.1% ± 13.2%	6.7% ± 6.2%
USSG Control (3-S)	197 ± 164	1.5% ± 2.7%	0	0	0.3% ± 0.5%	0	0	1.6% ± 2.7%	1.9% ± 2.4%	0.3% ± 0.5%
MPY Ground Zero	14 ± 9	8.9% ± 10.2%	0	0	0	0	0	8.9% ± 10.2%	0	10.4% ± 8.4%
MPY Impact Border	28 ± 12	7.9% ± 8.2%	0.05% ± 0.07%	0.3% ± 0.9%	0.9% ± 1.7%	0	0.8% ± 1.7%	10.4% ± 9.9%	14.6% ± 13.9%	4.7% ± 5.6%
MPY Control (1-N)	670 ± 437	2.2% ± 0.02%	0.05% ± 0.07%	0	0	0.17% ± 0.04%	0	2.4% ± 0.1%	3.1% ± 1.2%	0

**Fig 3:**
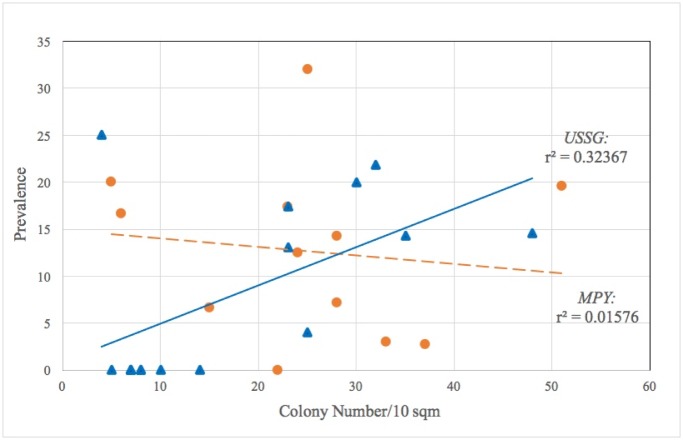
Simple regression of colony density on total disease prevalence within the two grounding sites. Ground-zero and impact-border zones pooled.

WS prevalence differed significantly between grounding and control sites (Table 3). However, the pattern we observed was not a simple ‘grounding site vs. undamaged control site’ difference; impact-border sites showed significantly more disease than either the ground-zero sites or the control sites, the latter two showing similar prevalence (Table 3). Neither WS nor total disease prevalence differed between the two grounding sites, suggesting similar long-term impacts of grounding damage. Physical damage differed between the zones and was lowest within the control sites. However, variability within the grounding sites was high, as some colonies had healed over time, making past injuries difficult to discern, and loose material had been transported by wave action; thus, differences were not significant (*p* > 0.05). Partial mortality, however, showed similar patterns with that of disease prevalence; significantly higher prevalence was observed within the impact border zones (Tables 2 & 3). We tested three potential drivers of WS prevalence patterns: host colony population density, persistent partial mortality, and physical injury. Final stepwise regression reduced to models with single predictor variables: Colony density explained 32% of the variation in total prevalence within the USSG site (*p* = 0.030), but only 1.6% within the MPY site (*p* = 0.697) (Fig 3); persistent partial mortality (PM) was positively correlated with active WS lesions, explaining 35% of the variation in WS prevalence in USSG and 31% in MPY. Further, colony density and partial mortality were correlated within the USSG site (*r* = 0.562, *p* = 0.0235), but not within MPY (*r* = 0.02, *p* = 0.949). In contrast, physical injury that was observable during our surveys was not significantly associated with active WS at either grounding (Table 3).

**Table 3:** Results of statistical analyses on patterns and drivers of coral-health impacts within the grounding zones and sites.

Comparison	Results
WS prevalence between ground zero, impact border, control	MPY: G = 29.35; *p*(G) = 6.65 *10(-6) USSG: G = 59.97; *p*(G) = 5.96*10(-13)
Total disease prevalence between ground zero & impact border	MPY: G = 5.43; *p*(G) = 0.0198; USSG: G = 13.139; *p*(G) = 0.00034
WS prevalence between ground zero, impact border, control, both sites	Zone: ANOVA F = 3.991; *p* = 0.0303 Grounding site: ANOVA F = 0.037; *p* = 0.8478 Scheffe post hoc: impact border > control = ground zero
Total disease prevalence between zones and grounding sites	Zone: ANOVA F = 7.234; *p* = 0.0028 Grounding site: ANOVA F = 0.248; *p* = 0.6222 Scheffe post hoc: impact border > ground zero = control
WS Prevalence MPY vs. USSG	G = 1.684; *p*(G) = 1.00
Partial mortality prevalence between ground zero, impact border, control, pooled grounding sites	Zone: ANOVA F = 8.755; *p* = 0.0012; Grounding Site: ANOVA F = 0.337; *p* = 0.717; Scheffe post-hoc: impact border>ground zero = control
Physical damage prevalence between zones, grounding sites pooled	ANOVA F = 3.0389; *p* = 0.063
Final regression model of WS prevalence on breakage, colony density and partial mortality as predictors, combined grounding sites	*WS prev* = 4.942 + 0.381(*Partial Mortality*); *n* = 28; *R*^2^ = 0.337; *F* = 6.665; *P* < 0.001
Final regression model of WS prevalence at USSG site.	*WS prev* = 4.942 + 0.381(*Colony Density*); *n* = 16; *R*^2^ = 0.316; *F* = 5.900; *P* < 0.023
Final regression model of WS prevalence at MPY site.	*WS prev* = 5.044 + 0.453 (*Partial Mortality*); *n* = 12; *R*^2^ = 0.415; *F* = 5.704; *P* < 0.024

**Fig 4:**
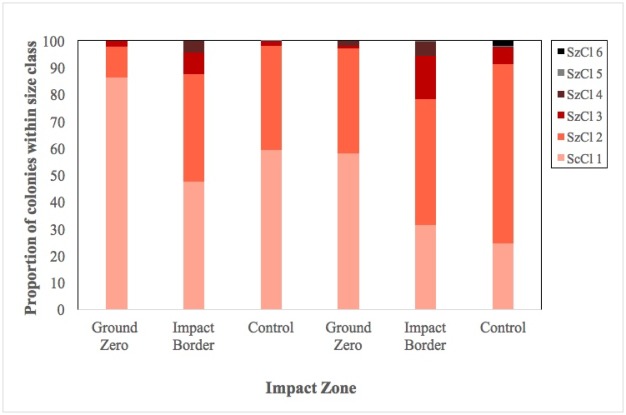
Proportional size-class distribution of coral colonies within the *Min Ping Yu* and *USS Guardian* grounding sites. Size classes are based on maximum colony diameter: 1: 1–10 cm; 2: 11–30 cm; 3: 31–60 cm; 4: 61–100 cm; 5: 1–2 m; 6: ≥2 m.

The 2015 assessments showed a profound effect of the grounding events on the coral community size structure that were pronounced two years later, and which may have influenced disease patterns. Ground-zero zones were almost completely reduced to a few colonies of the smallest size classes, largely post grounding recruits <5cm in diameter (Fig 4). Likewise, colony numbers were higher within impact-border zones, but still much lower than that of their respective sites, and with few to no larger colonies. Within the MPY site, the number of colonies per size class was an order of magnitude larger within the undamaged control site (33.5 col m^-2^) than within the ground-zero (1.5 col m^-2^) or the impact border (2.2 col/m^2^). A similar pattern, though not as profound, was observed within the USSG site (9.9 col/m^2^, 0.8 col/m^2^, and 3.1 col/m^2^, respectively). While this may be partly explained by influences of differing exposure regimes between the grounding and respective control sites, reduction in the range of size classes points to a prolonged effect of the grounding incidents.

## Discussion

This study provides the first report, to our knowledge, of long-term impacts of coral disease and physical injury from multiple ship groundings on a pristine coral reef. The groundings that occurred in early 2013 caused massive acute physical damage to corals in the reef zones with the densest coral cover (shallow forereef to reef crest), which was apparent two years after the events. Evidence of damage was obvious not only from the loss of reef structure, but also visible as persistent partial mortality and coral disease. Disease prevalence within pristine control sites and ground zero zones was lower than in most other sites surveyed within the Philippines, but similar to values reported from well-managed MPAs in the Central Visayas (Table 4). Conversely, disease prevalence, partial mortality and physical injury were highest in the impact border zones and disease prevalence, in particular, was among the highest reported in the country (Table 4). Our results suggested that these patterns were associated with prevalence of partial mortality and physical injury within these zones.

**Table 4:** Reports of coral-disease prevalence from studies completed within the Philippines; *n* refers to number of sites.

Survey Location	n	Mean Total Disease Prevalence (+/-SD)	Source
Central Visayas	4	11.6 +/- 2.8	[34]
Lingayen Gulf	4	5.1 +/- 1.4	[34]
SE Negros Island	15	23.9 +/-18.8	[33]
Tañon Strait	7	16.3 +/- 21.1	[33]
Bohol Sea	3	5.1 +/- 6.2	[33]
Palawan Island	2	4.9 +/- 1.3	[33]
Central Visayas Marine Protected Areas	5	2.8 +/- 0.9	[40]
Central Visayas paired fished	5	4.5 +/-1.2	[40]
Bantayan Island Marine Protected Areas	4	7.13 +/- 3.41	[41]
Bantayan Is. fished sites	5	2.26 +/- 2.27	[41]
Tubbataha control sites	2	3.5 +/- 1.8	This study
Ship grounding ‘ground-zero’ sites	2	1.9 +/- 1.4	This study
Ship grounding ‘impact-border’ sites	2	14.8 +/- 4.5	This study

### White syndrome dynamics

A grounding event causes immediate and drastic physical damage to corals and mobile debris can continue to injure and abrade corals long after the initial damage. Two years after the grounding events, colonies within the impact border sites showed partial mortality and disease prevalence five to 15 times higher than in either ground-zero zones (dominated by post-grounding recruits) or undamaged control sites. Further, these metrics were significantly positively correlated (Table 3), suggesting that a majority of lesions arose as a result of grounding events and have persisted over time. We saw no other potential cause of partial mortality during our surveys, as lesions did not resemble those caused by Crown-of-Thorns starfish (which were not observed), parrotfish, or *Drupella* predation, or bleaching mortality. Interviews with resident rangers and monitoring personnel did not reveal any other event which may have explained the patterns of mortality we observed.

The possibility of an undocumented WS outbreak in the months following grounding damage should not be discounted and would account for high prevalence of both persistent partial mortality and acute WS. Infectious disease entering a naïve but susceptible host population (as suggested by the very low disease prevalence within our control sites) may exhibit high initial infection levels leading to an exponential increase in infected hosts, followed by a transition to a chronic infection state typically manifested by lower but persistent disease prevalence [42]. Corals surviving a disease may thus either recover and regrow tissue, or persist with chronic, low-level infections. Re-infection could potentially occur if conditions are favorable for the pathogen or stressful to the host [43]. This phenomenon has been described for *Montipora* WS, which manifests both acute and chronic phases associated with different invading organisms that result in similar rates of colony mortality and recovery [44,45]. In the case of our grounding sites, site inaccessibility prevented us from monitoring reef responses in the months following the grounding events, which would have allowed us to detect an outbreak. Our surveys did detect active WS infection associated with many partial mortality lesions, but not all WS lesions were bordered by areas of partial mortality (suggesting new sites of infection) and not all partial mortality lesions showed active WS. Further, while physical injury was significantly higher within impact border sites than within other zones, injury was not correlated strongly with disease patterns two years post-grounding. Visible signs of tissue resheeting on colonies that had been toppled, broken or fragmented, suggested that many injuries may have healed and initial prevalence of physical injury was undoubtedly much higher than that recorded two years after the incidents. Together, these observations suggest multiple and dynamic causes of the mortality we observed in the impact border zones which are linked with the initial damage caused by the grounding incidents.

White syndrome may, thus, be persisting among older colonies within the impact border zone. This can be facilitated by interactions between the stress of physical injury and high coral colony density, which was a positive driver of white syndrome prevalence within the USSG grounding site (Fig 2), suggesting a density-dependent infection [46,47]. Longer-term trajectories of white syndrome impacts on this population may be influenced by recruiting juvenile colonies. Recruits entering into this population may be at risk due to the high prevalence of WS. Conversely, they may be less susceptible than the existing population and remain unaffected in the absence of further stress, thus providing a means of recovery. Interestingly, the MPY site did not evince a similar relationship between host density and disease prevalence (Fig 2), which suggests that either coral density is below a threshold level that facilitates disease spread or that there are other factors driving disease patterns at this site.

### Healing and regeneration processes

In corals, tissue regeneration involves resheeting over bare skeleton, which otherwise becomes rapidly colonized by fouling communities which impedes future resheeting. Roff et al. [48] noted that physical injuries in corals trigger resource allocation toward the wound to initiate healing, while lesions caused by a microbial agent trigger the opposite effect; resources are allocated away from a disease lesion in an apparent attempt to starve the diseased tissue. Thus, while acute physical trauma may stimulate healing, it also creates conditions favoring subsequent bacterial infection. As regeneration rate per unit area of colony surface is negatively correlated with lesion size [49,50] larger lesions may be at increased risk of pathogenic infection. Thus, large lesions may persist over time [51] and become recruitment surfaces for other organisms. Reinfection has been reported for black band disease [52] and has been observed on WS infected colonies in Guam (Raymundo unpubl. data).

### Recovery processes

Long-term impacts of the grounding events within these pristine atolls will be dictated by the success of recovery processes. The ground-zero zones were largely devoid of corals but recruitment was evident and recruits were healthy. Recovery trends within the USSG site, in particular, indicate that slow-growing, long-lived corals began to play a significant role in coral cover increase within four years post-grounding. However, the MPY site has not followed a similar trend. It is interesting to note that, despite abundant large coral skeletal fragments providing more colonizable space than the surrounding sand substrate, recovery has been slow to non-existent. Persistent unconsolidated rubble has buried, abraded and injured recruits and small colonies, which is preventing recovery. Unstable substrates created by physically destructive events persist over time simply because natural recovery via recruitment is prevented [53,4].

Given the protected status of the TRNP, the role of fish in recovery is likely to be significant. Within grounding sites, substrates were devoid of macroalgae and showed only sparse turf, suggesting high herbivory. While we observed recruit mortality from corallivory, we hypothesize a greater positive impact on recovery via herbivory. The link between herbivory and benthic resilience is well-established; herbivory controls algal abundance and protects high quality recruitment substrates, thus facilitating coral growth and recruitment [54,55]. Wilson et al. [56] described a complex relationship between coral decline and fish community change. Where coral mortality did not result in the loss of reef structure, fish habitat was preserved and fish declines were not observed. However, a loss of structure, such as with a grounding event, can lead to algal blooms (as reported by [57]), with subsequent changes in the fish community, resulting in a loss of coral-dependent species and increase in algal feeders. We predict that this effect will diminish over time, as fish will recruit from surrounding healthy reef, facilitate restoration of the previous community. There were no macroalgal or cyanobacterial blooms reported post-grounding either by monitoring teams or rangers within the Tubbataha grounding sites.

### Climate change, bleaching and the future

The TRNP is one of the best-managed marine protected areas within the Philippines and, arguably, the world. Anthropogenic impacts, at present, have been minimized by a resident ranger patrol presence, highly seasonal access to the site, limited dive boat anchorage, a strong presence by scientific and management agencies, and a well-organized and committed management staff (the Tubbataha Management Office). However, local management efforts may not be sufficient to maintain its current near-pristine condition in the face of global climate change. It is likely that warming events, and the stress they place on corals, will have a deleterious effect on the reef’s ability to recover from damages such as grounding events in the future. It is reasonable to assume disease outbreaks could occur in reef areas most affected by seasonal temperature increases, with the threat to corals compounded by physical damage from dive operations. Associations between temperature-induced bleaching and disease outbreaks have been quantified for white syndrome [58,59] and black band disease [60,61,52], suggesting that in an era of warming climate, certain coral diseases may increase in prevalence and/or severity.

Recent events may provide a view of future trends. In 2014, the Park underwent two bleaching stress threshold events over a three-year period, with one of those events constituting severe stress (“stress threshold” defined as DHW of 4°-C weeks; “severe stress” defined as DHW of 8°-C weeks; [62]). The timing of this event corresponded with a loss of coral cover that was detected in our 2015 fixed plots in the USSG grounding and control sites (Table 3), but not in our randomly-sampled monitoring stations [2]. However, we note that the 2014 monitoring occurred prior to the bleaching season, so subsequent bleaching and/or a disease outbreak during that year would not have been detected until the following year as either partial mortality or loss of coral cover. This event resulted in a setback that delayed recovery and, if warming events increase in frequency as predicted, this could potentially result in a failure to recover to the pre-grounding state.

Historically, TRNP experienced bleaching stress in the 1998 ENSO event [63], but between 1998 and 2014, there is little information about bleaching severity, as monitoring and active management had not yet begun. Using Degree Heating Weeks (DHW) historical temperature data to assess bleaching stress, Heron et al. [62] listed Tubbataha as one of the few World Heritage Sites historically not severely affected by bleaching events over the past three decades.

However, future scenarios under both Representative Concentration Pathways (RCP 4.5 and RCP 8.5) predict severe bleaching events twice per decade by 2037 for RCP 4.5 and by 2030 for RCP 8.5 [62]. Thus, reducing CO_2_ emissions from the current track (RCP 8.5; the “business-as-usual” scenario) to RCP 4.5 provides the TRNP with an additional seven years of time to acclimatize to increased temperatures. However, this small grace period may not improve conditions for the most susceptible species. As with other coral reef hot spots of exceptional quality, drastic reductions in CO_2_ emissions are urgently needed to stave off potentially huge losses in biodiversity and coral reef function. Managing local sources of additional stress is currently thought to be a strategy to reduce—but not eliminate—the impacts of climate change [64]. In Tubbataha, localized stress is mainly derived from tourism operations, which are concentrated during the warm summer season. Expanding such operations should, therefore, be accompanied by determination of a diver carrying capacity [65], establishing a monitoring program for dive sites to track changes in coral health associated with dive operations, and training of divers in minimizing physical damage to reef organisms [66]. In addition, maintaining the current level of protection, minimizing other sources of physical damage, and ensuring high water quality are all essential to protection of this unique heritage site.

## Supporting information

S1 Table Raymundo, Licuanan, Kerr. Recovery Plots.xlsx—Spreadsheet for live coral percent cover by plot and year.
(XLSX)

S2 Table Raymundo, Licuanan, Kerr. Coral Disease.xlsx—Spreadsheet for records of coral disease by site, species, and size class.
(XLSX)

S1 Fig Small and large rubble patches within the Min Ping Yu impact border zone. A. small rubble patch (ground zero of hull impact scar to the left and large rubble in the foreground); B. large rubble patch (ground zero of hull impact scar to the right). Photo credits: W. Licuanan.
(JPG)

S2 Fig Examples of diseases of corals in Tubbataha Reef Nature Preserve. A. White syndrome; B. Growth anomaly; C. Skeletal eroding band disease; D. Brown band disease; E. Ulcerative white spot disease; F. Black band disease. Photo credits: L. Raymundo.
(JPG)

S3 Fig Examples of physical injuries to corals. A. Colony fragmentation, which subsequent reattachment (at red stars); B. Colony abrasion/shearing; C. Colony fracture. The original colony is identified by the yellow circle; red stars identify fragments of the colony that were fractured from the colony on impact with the ship hull. Photo credits: L.J. Raymundo.
(JPG)
