# Correction: Adding insult to injury: Ship groundings are associated with coral disease in a pristine reef

**DOI:** 10.1371/journal.pone.0207078

**Published:** 2018-11-01

**Authors:** L. J. Raymundo, W. Y. Licuanan, A. M. Kerr

The second author’s name is spelled incorrectly. The correct name is: W.Y. Licuanan. The correct citation is: Raymundo LJ, Licuanan WY, Kerr AM (2018) Adding insult to injury: Ship groundings are associated with coral disease in a pristine reef. PLoS ONE 13(9): e0202939. https://doi.org/10.1371/journal.pone.0202939
