# Supplementary figures and images for: Adding insult to injury: Ship groundings are associated with coral disease in a pristine reef
Source: PLoS One. 2018 Sep 12;13(9):e0202939. doi: 10.1371/journal.pone.0202939 (PMC6135382; doi:10.1371/journal.pone.0202939)

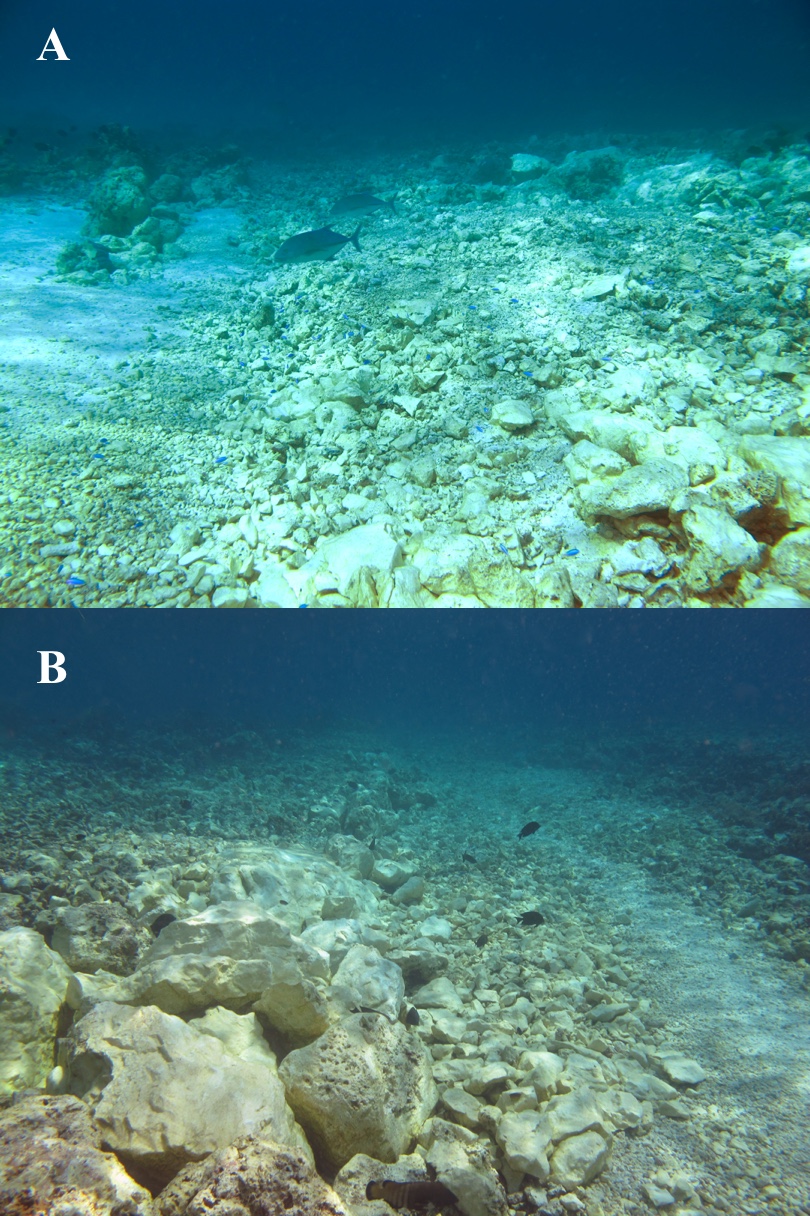

Supplement: S1 Fig — A. small rubble patch (ground zero of hull impact scar to the left and large rubble in the foreground); B. large rubble patch (ground zero of hull impact scar to the right). Photo credits: W. Licuanan. (JPG) [file pone.0202939.s003.jpg]

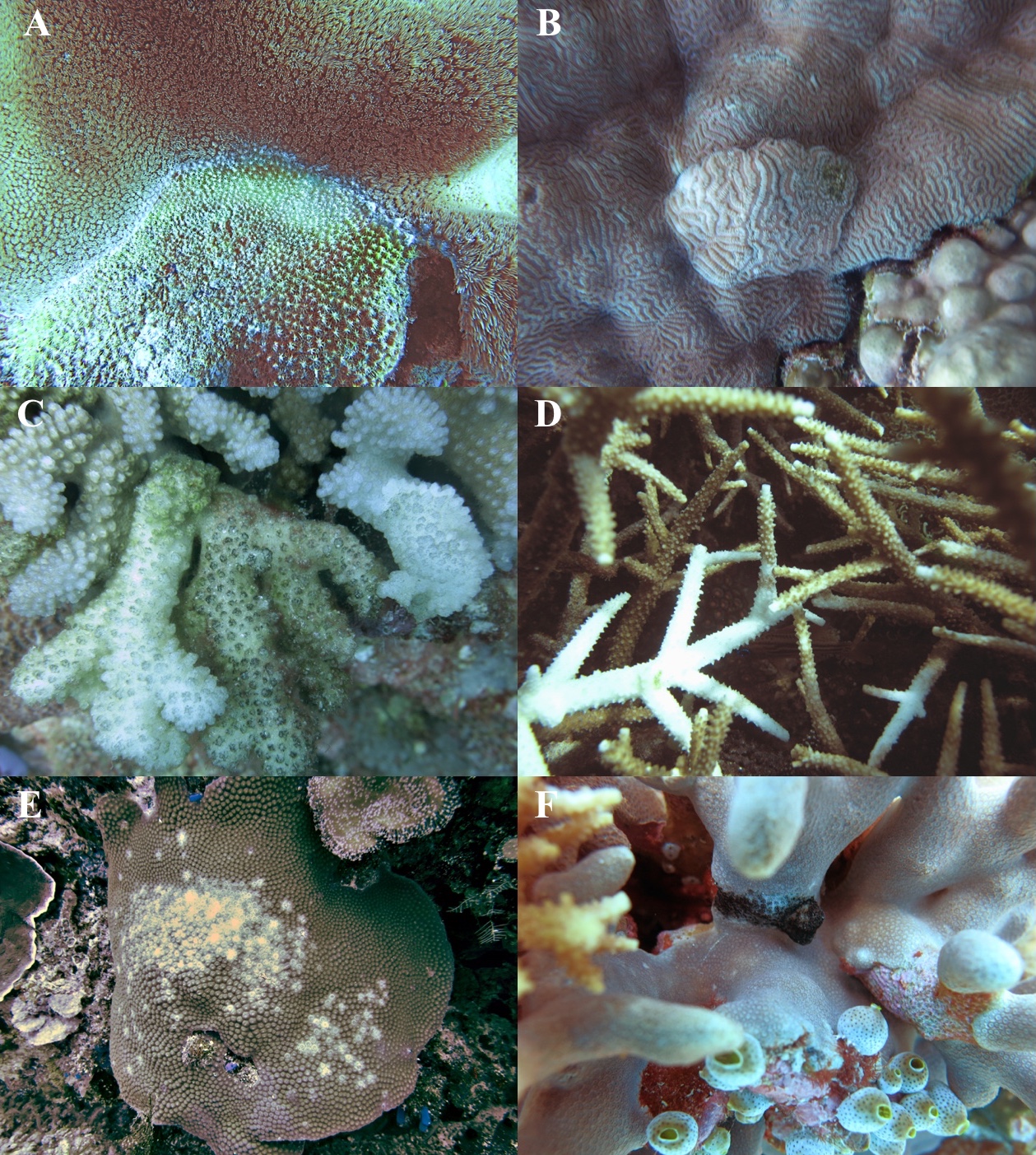

Supplement: S2 Fig — A. White syndrome; B. Growth anomaly; C. Skeletal eroding band disease; D. Brown band disease; E. Ulcerative white spot disease; F. Black band disease. Photo credits: L. Raymundo. (JPG) [file pone.0202939.s004.jpg]

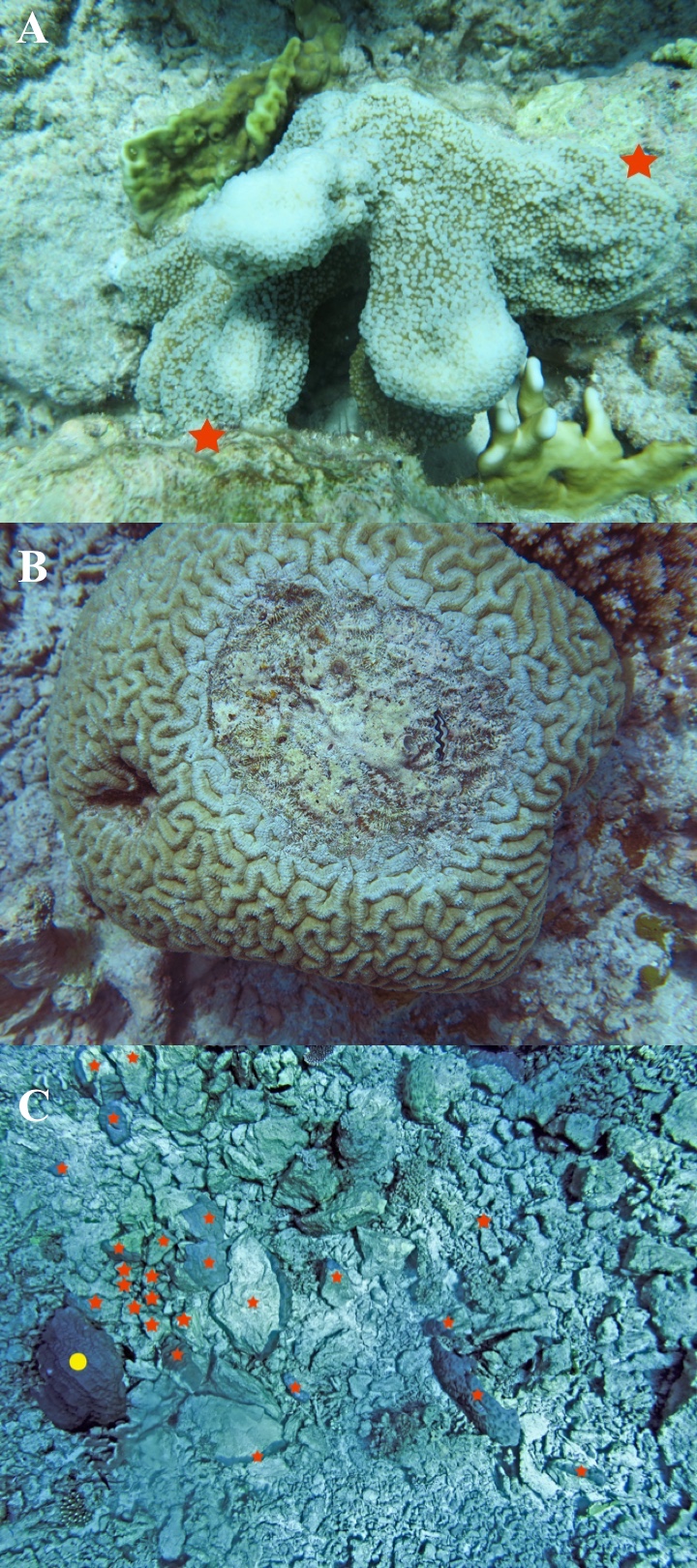

Supplement: S3 Fig — A. Colony fragmentation, which subsequent reattachment (at red stars); B. Colony abrasion/shearing; C. Colony fracture. The original colony is identified by the yellow circle; red stars identify fragments of the colony that were fractured from the colony on impact with the ship hull. Photo credits: L.J. Raymundo. (JPG) [file pone.0202939.s005.jpg]
